# Improving Survivors’ Quality of Life Post-Treatment: The Perspectives of Rural Australian Cancer Survivors and Their Carers

**DOI:** 10.3390/cancers13071600

**Published:** 2021-03-30

**Authors:** Kate M. Gunn, Ian Olver, Xiomara Skrabal Ross, Nathan Harrison, Patricia M. Livingston, Carlene Wilson

**Affiliations:** 1 Cancer Research Institute, University of South Australia, Adelaide 5001, Australia; xioyen@gmail.com (X.S.R.); nathan_harrison@rocketmail.com (N.H.); 2 Department of Rural Health, Allied Health and Human Performance, University of South Australia, Adelaide 5001, Australia; 3 Flinders Centre for Innovation in Cancer, College of Medicine and Public Health, Flinders University, Adelaide 5042, Australia; C.Wilson2@latrobe.edu.au; 4 Cancer Council SA, Adelaide 5067, Australia; 5 School of Psychology, University of Adelaide, Adelaide 5001, Australia; ian.olver@adelaide.edu.au; 6 Deakin University, Melbourne 3125, Australia; trish.livingston@deakin.edu.au; 7 LaTrobe University, Melbourne 3086, Australia

**Keywords:** cancer, oncology, survivor, survivorship, rural, remote, post-treatment, quality of life, psychosocial, support

## Abstract

**Simple Summary:**

Existing programs to support cancer survivors post-treatment tend to be delivered face-to-face, reducing their accessibility to those living in rural and remote locations. Additionally, little is known about the acceptability of urban-developed survivorship care programs among rural cancer survivors who may have unique values and different attitudes towards help-seeking. The purpose of this study was to explore the experiences of cancer survivors who return to their rural communities upon completion of active treatment, and to identify the challenges these survivors experience in engaging with quality of life-related support services. The findings of this study will inform the design and development of new interventions, or modification of existing interventions, to better meet the preferences and needs of rural survivors. Identifying the specific challenges and intervention preferences of rural cancer survivors will help to ensure they benefit as much as urban survivors, from efforts to improve post-treatment quality of life.

**Abstract:**

The transition from urban centres back to rural and remote communities can be challenging for rural cancer survivors after treatment. This study aimed to (a) provide deeper understanding of the experiences of rural survivors who have completed active cancer treatment and returned to their rural communities, and (b) determine strategies to re-orient existing services or develop new interventions to more appropriately meet rural survivors’ service preferences and needs. Semi-structured interviews were conducted with 22 adults (64% female) who lived outside of a metropolitan area and had completed active cancer treatment (n = 13), were the carer for a rural/remote cancer survivor (n = 6), or were both a survivor and carer (n = 3). Thematic analysis was conducted to identify dominant themes in the qualitative data. A range of physical, psychological and practical challenges that impact quality of life among rural survivors post-treatment were found. These challenges appeared to be compounded by a lack of trust in local rural healthcare services and a lack of clear post-treatment pathways to quality of life-enhancing support services. Acceptable strategies to overcome barriers included nurse-led, telephone-based, or face-to-face interventions, initiated and continued by the same service provider, and that included support to manage emotional challenges associated with post-treatment survivorship. The findings will inform the design of interventions to better meet rural cancer survivors’ post-treatment support needs.

## 1. Introduction

Approximately one third of people affected by cancer in Australia live outside major metropolitan centres in ‘rural’ areas [1]. Rural cancer survivors not only face the challenges that are associated with a diagnosis of cancer in any setting, but a number of additional stressors; for example, the distances they have to travel for treatment [2,3]. Distress and poor quality of life is complicated for rural survivors by poorer treatment outcomes [4,5], limited levels of access to information [6], and multiple barriers to accessing support [7].

The completion of treatment and transition from urban treatment centres back to rural communities can be challenging for rural cancer survivors. A small number of qualitative studies among rural breast [8,9] and haematological [10,11,12] cancer survivors have found that upon completing treatment and returning home, they often report feeling isolated, fearful and fatigued. Losing support from specialist treatment teams at this time can also be challenging, particularly when they perceive their local health services as lacking knowledge about cancer [10]. More broadly, recent quantitative research has highlighted that *rural* Australian cancer survivors (with any cancer diagnosis) are more likely than *urban* cancer survivors to be overweight and to not engage in any physical activity [13]. These patterns are also likely to be true for the broader rural population, but are particularly problematic for cancer survivors as they significantly impact upon their quality of life and increase their risk of cancer recurrence [14,15]. People living in remote areas of Australia are 35% more likely to die within five years of a cancer diagnosis than those living in large Australian cities [5] and although survival is generally improving, disparities in outcomes between rural and urban cancer patients are growing [16,17]. Therefore, finding contextually and culturally appropriate ways to increase rural cancer survivors’ engagement in health-promoting behaviours such as exercise and ongoing surveillance is an important endeavour [13], as is finding appropriate supportive care strategies to improve their quality of life [11,18,19,20].

Several evidence-based programs to support cancer survivors improve their quality of life post-treatment have been developed and these have been shown to be efficacious in general cancer populations [21]. However, as many are delivered face-to-face, their accessibility by rural survivors is limited, and the acceptability of urban-developed survivorship care programs by rural cancer survivors, who are known to have unique values [22] and different attitudes towards help-seeking [23], is not well understood. There is also little known about rural cancer survivors’ willingness to engage in self-management strategies designed to improve their quality of life [24]. Online programs are increasingly being developed to assist cancer survivors with post-treatment quality of life issues such as fear of cancer recurrence and sleep disturbance, but a recent review found that, although rural survivors have much to gain from the delivery of survivorship interventions online, most research is focused on online interventions designed for urban breast cancer survivors in the United States [25]. They concluded that “research is needed on interventions that take into account the diversity of cancer types, unique environments, attitudinal barriers to engagement, and psychosocial challenges (e.g., isolation) that rural survivors may face” [25] (p. 9).

To address these knowledge gaps and inform future intervention work, there is a clear need to determine the types of strategies that may help rural cancer survivors adopt healthy lifestyles, manage treatment side effects, reduce their risk of cancer recurrence, and, more generally, improve their quality of life. Further, as carers play a pivotal role in assisting cancer patients and survivors with the management of their disease and treatment [26]—particularly in rural settings where access to professional services is more limited and they may be responsible for assisting with transport—understanding what is feasible from their perspective is also important.

Consequently, this study aimed to explore the perceptions of rural survivors and carers in order to: (a) provide deeper understanding of the experiences of rural people who have completed active cancer treatment and returned to their rural communities; and; (b) determine methods through which existing post-treatment quality of life-enhancing intervention services could be re-orientated or new interventions developed, to appropriately meet rural survivors’ preferences and needs.

## 2. Materials and Methods

### 2.1. Participants

To be eligible to participate in the study, individuals had to have been diagnosed with cancer and had completed active treatment, or be the carer or main support person of someone who had been diagnosed with cancer and had completed active treatment. They also had to be aged 18 years or older, fluent in English, capable of giving informed consent, and reside outside metropolitan areas (in an accessible, moderately accessible, remote, or very remote region of Australia) according to the Accessibility/Remoteness Index of Australia (ARIA; [27]). Cancer survivors were ineligible to participate if they had been diagnosed with a non-melanocytic skin cancer (or another non-reportable type of cancer), were still undergoing active cancer treatment (e.g., radiotherapy or chemotherapy), or they did not feel physically or psychologically capable of being interviewed for approximately one hour. Carers were ineligible to participate if the person they cared for was still receiving active cancer treatment or had lived for less than 18 months after completion of treatment.

### 2.2. Procedures and Materials

Ethics approval was granted by the Flinders University Social and Behavioural Research Ethics Committee and the University of South Australia Human Research Ethics Committee (Application ID: 0000035930). Interviewees expressed an interest in participating in response to media articles, posters displayed in rural General Practitioner (GP) practices or subsidised accommodation facilities for rural cancer patients, and notices shared by rural cancer support groups. Semi-structured, face-to-face interviews were conducted by a female researcher (KG). KG is a registered clinical psychologist with a rural background and prior experience interviewing rural people affected by cancer for research purposes. She did not have an established relationship with any of the study participants. Cancer survivors and carers were interviewed either separately or together, depending on participants’ preferences. Confidentiality was explained and written informed consent obtained from all participants prior to participation in an interview. Open-ended and probing questions were asked based on the relevant literature. Topics covered in the interviews are outlined in Table 1. Interviews were audio-recorded and transcribed verbatim. Data collection ceased when data saturation was reached.

### 2.3. Analyses

An essentialist/realist methodology was employed, using an inductive approach, to analyse the qualitative data. This meant that themes were closely linked with the data, situating thematic meaning at the semantic or surface level of the data. Dominant themes were identified using Braun and Clarke’s Thematic Analysis method [28]. Themes were defined as common ideas present within the data that were relevant to the objectives of the study. Data were independently coded by KG and XSR. After discussion, 100% agreement was reached between the two researchers on resulting themes and subthemes. The Consolidated Criteria for Reporting Qualitative Research (COREQ) was used to aid reporting of the methods and results [29].

## 3. Results

Twenty-two participants (64% female) agreed to take part in semi-structured, face-to-face interviews. Participants were purposively sampled to ensure representation of diverse educational backgrounds, roles (survivor, carer or both), medical histories (cancer diagnosis and time since treatment completion), genders and levels of remoteness (according to ARIA categories [27]). Four participants were from ‘inner regional’, 11 from ‘outer regional’, four from ‘remote’ and three from ‘very remote’ ARIA regions. Thirteen participants reported a history of cancer, six reported a history as a supporter of someone with cancer, and three reported both a personal history of cancer and as a supporter. All participants reported disease status as ‘no activity’. Fifty per cent of participants were considered short-term survivors (i.e., they or the person they cared for had completed treatment in the two years prior). Fourteen participants (63.6%) reported that they currently had private health insurance. Additional demographic and cancer history characteristics are reported in Table 2.

**PART A: Post-treatment challenges that impact upon quality of life**

Four overarching themes and several subthemes relating to post-treatment challenges that impact upon rural survivors’ quality of life, emerged from the data.

### 3.1. When Treatment Is Over, It Is Not All Over—Quality of Life Is Often Not Restored

Participants highlighted that the completion of treatment does not signal the end of the negative impact of cancer on their lives. Reasons for this are detailed in the subthemes below.

- Participants reported experiencing a number of ***long-term physical side effects after cancer treatment that affect their quality of life***, for example, sexual and fertility issues, fatigue, side-effects from medication, comorbidities (e.g., diabetes) and post-operative issues that require ongoing management (e.g., drains, swelling, lymphedema).
  *“I started having the joint issues probably six months after… then I spent two years at home. I couldn’t walk… Then I got back to work… It’s hard, because everything still keeps going wrong. You get over one thing… then something else happens… everything seems to be going wrong again now—bleeding front, back. You’ve got diabetes… so you just get a bit frustrated.”* (Male, prostate, long-term survivor, #7)

b. Moreover, participants reported that ***new psychological challenges*** (e.g., fear of recurrence, decreased control, feeling depressed, survivor guilt) ***arise*** at this time.
  *“I [felt] more confident while I was doing treatment than when I came home, because I felt like I was doing everything I possibly could. Then you’d step off.”* (Female, breast, short-term survivor, #11)
  *“Every day. Every day you wake up and the big C is right there.”* (Female, breast, short-term survivor, #14)

c. Participants expressed ***frustration due to unmet expectations about their return to pre-cancer quality of life*** after treatment. This could be compounded when it was not recognised by others.
  *“You know, people just don’t get it… it’s like toughen up, you know, you’ve had your treatment, you’re done… they don’t get that um it actually has physically affected his body.”* (Female, carer of long-term sarcoma survivor, #5)

d. Challenges may also be associated with related ***reduced levels of independence*** when managing daily activities.
  *“There are certain aspects of memory—like if I put something away, I nearly need to write myself a note where I put it.”* (Male, brain and prostate, short-term survivor, #22)

e. Feelings of ***isolation***, particularly from treating teams, but also from peers, family and friends, were also prevalent.
  *“… the distance and the isolation.”* (Female, cervical, long-term survivor, #16)
  *“Um they [urban patients] haven’t had to, yeah, do the whole move thing and then go home again and then leave all the friends that you’ve then made in [the city].”* (Female, carer of long-term sarcoma survivor, #5)
  *“Yeah, so when you’re in treatment, everyone’s right close by you, holding your hand and guiding you… through every step… And, you know, very close with you and hands on, whatever. But once-once you’ve finished your treatment, it’s-it’s let go virtually and said, oh off you go, see you later, and nearly push you out the door, you know, and-and see you later. Well that’s what-a bit how it felt to me, you know… I didn’t know where to go, what to do, I was lost, basically is how I felt.”* (Male, testicular, short-term survivor, #4)

f. Participants also reported they were ***lacking a clear post-treatment care pathwayand information***. They consistently expressed confusion about the specialist they should be seeing for medical follow-ups or to address new medical concerns. They also reported receiving conflicting information, or no information at all, about the post-treatment strategies they could employ to maximise their quality of life.
  *“It’s a consequence of actually not doing the right thing [lifting heavy boxes after treatment], but I still to this day don’t actually believe that I had enough instructions as to what not to do. Yeah, they just said yeah, look after yourself.”* (Female, colorectal/lymphoma short-term survivor and carer of long-term prostate cancer survivor, #21)

### 3.2. Lack of Confidence in Local Rural Health Services’ Ability to Help Them Address Post-Treatment Quality of Life and General Health Issues

Participants reported lacking confidence in the ability of and resources available to their rural health services, to help them address their post-cancer treatment quality of life and medical issues. The reasons for this are outlined in the subthemes below.

- ***The lack of continuity in rural general practitioners (GPs) is problematic.***
  *“Don’t have the same GP. We’re in the country situation where you ring up and you get who you get. I had a really lovely GP that actually diagnosed my breast cancer—and she’s left.”* (Female, breast, short-term survivor, #11)

b. ***Long waiting times to see GPs*** was also commonly reported.
  *“You can’t see a GP here for life or money, for a week.”* (Female, breast, short-term survivor, #14)

c. Several participants reported ***difficulty understanding their GP.***
  *“I had trouble not understanding her language so much as understanding her way of explaining things. Yes, she was from overseas somewhere and that doesn’t matter, a lot of our doctors are now. So yes, that just makes it that little bit more difficult”*(Male, prostate, long-term survivor, #12)

d. More general concerns about the ***quality*** of local healthcare were also evident.
  *“I don’t like the doctors here. I’ve had the same GP in [city] because I used to live there so I just kept them. [The local doctors] often look things up while you’re in there and I would rather drive an hour and a half and know that I’m getting the care that I need… You just can’t rely on them. I think my options are better elsewhere.”* (Female, cervical, long-term survivor, #16)

e. The ***limited scope*** of medical services provided by rural hospitals is also a source of concern for this group.
  *“… came home and had a bowel obstruction… I don’t know how many people have spoken to you about the [local] hospital. Well they won’t deal with… anything. Anything. Anything. Just forget it. So they [flew] me out…”* (Female, breast, short-term survivor, #14)

f. The ***lack of availability*** of relevant local services to improve quality of life was also noted:
  *“So, got home and it was like it hadn’t happened… It probably would have been helpful to have someone and maybe potentially not in Adelaide because everything’s in Adelaide… if there was someone, not necessarily in [this town], but in this area that I could have spoken to because like I said, the biggest thing I think I had was life just went back to normal and I was just sitting here thinking it’s not for me.”* (Female, cervical, long-term survivor, #16)

g. Participants also expressed a ***lack of information and direction on what is available and how services may be able to help*** them improve their quality of life.
  *“… and Dr [name of doctor] says, “What are you worried about?” “Well, I don’t know. I worry about everything”. So, he says, “Do you want to take tablets?” I said, “No. I don’t want tablets, but I’ve got to stop worrying” but it’s so easy to say that and do it is not as easy … I don’t know. Where would I go? Dr [name of doctor] should tell me where to go.”* (Male, prostate, long-term survivor, #9)

### 3.3. Challenges with Returning to Metropolitan Centres for Ongoing Cancer Surveillance and Follow-Up Care

Returning to metropolitan cancer centres after completion of cancer treatment can also be challenging for the reasons outlined below.

- It can require significant amounts of ***time away from work and/or family***. For example, a ten-minute check-up may take two-three days out of a rural survivors’ week (compared to just a morning for someone living close to treatment facilities).
  *“So and when you’re working you’ve got to get time off of work. You’ve got to get someone to look after your other kids… So it’s not, not an easy, yeah... sometimes they forget that.”* (Female, carer of long-term sarcoma survivor, #5)

b. Participants also reported the ***inconvenience of******medical appointment scheduling practices*** in metropolitan centres that do not consider the residential location of the survivor. For example, not being able to book all appointments on the same day or attending early morning appointments that require them to travel the day before (and find accommodation) were described as vexing. Last minute appointment cancelations lead to challenges rearranging travel logistics and time out of work.
  *“They say, just come back if there’s anything, you need anything. Yes, but it takes me a whole day and then you’ve got to make an appointment and you often can’t get appointments at times that suit you and things just aren’t quite as easy… in terms of going back or if I needed someone to come with me. My parents still work and my husband was straight back into work so he can’t just have time off to go and do things again…”* (Female, cervical, long-term survivor #16)
  *“It’s the logistics of doing it. It’s all right—when they say you’ve got a nine o’clock appointment, you’ve got to go the day before. Things like that. For us, it’s oh my God, where do you stay? Where do you park? The city is so busy.”* (Female, breast, short-term survivor #18)
  *“I had to have an operation here earlier in the year, actually it was supposed to have been done last year late and went all the way to Adelaide, spent all day in the hospital and then it was cancelled. Now that is a nightmare. Whereas if you were in the city it’s not quite so bad, you haven’t got so far to go home and then go back again.”* (Male, prostate, long-term survivor, #12)

c. For many, returning to treatment centres for follow-up care comes with significant ***financial costs***. Despite schemes to partially reimburse rural patients for travel-related expenses, spending time away from home and work is costly in other ways too.
  *“But it’s still—you know, I’m a casual worker now and I don’t get a sick day or…you know it’s hard to arrange time off. Someone says, oh I want you on next Friday…oh shit, sorry, I can’t make it ‘cause I gotta go to Adelaide, well they don’t say, well come Monday… you not only don’t get sick leave, you’re losing money as well.”* (Male, testicular, short-term survivor, #4)
  *“Because we’re self-employed we had the financial hit of all the travel and accommodation where we had to pay, plus the time issue. Whereas most people can get sick leave or whatever.”* (Female, breast, short-term survivor, #11)

### 3.4. Most Post-Treatment Quality of Life-Focused Support Is Provided by Family, Friends, Nurses (Visiting and via Structured Telephone-Based Support Programs) and Support Groups

- ***Practical lay support is especially highly valued, due to fatigue and other treatment-related side effects*** which make attending to practical tasks challenging.
  *“I had a good network probably around me… Oh, mowed the grass, wood for the fire, filled the woodshed, stacked it, chopped it etc., just practical things I suppose. Yeah. That more physical stuff I suppose…”* (Female, breast, short-term survivor, #18)

When this assistance was not available, participants reported feelings of isolation and low mood. Although not explicitly stated by participants, there was evidence to suggest that lay practical support was not only of practical value to this group, but also helped emotionally, by making them feel as though their challenging experiences were acknowledged, and that they were cared for and not alone.
  *“I’ve heard of other people who have gone through surgery and friends have taken them food and all that sort of stuff. No one did a damn thing for me, so it was that lack of caring—I didn’t feel like anybody really cared when I came back, because they didn’t show anything. They might talk to you in the street and ask, but to actually do anything, and some of the things that I couldn’t do that I shouldn’t have been doing but I did do because no one else was here to do it for me—so there was that was where I felt it really strongly.”* (Female, lymphoma, short-term survivor, #15)

b. The ***assistance delivered by nurses to survivors in their homes was highly valued.*** Nurses perform a wide range of tasks in these settings, for example attending to wounds and assisting with showering.
  *“The country nurses were absolutely brilliant. They are salt of the earth. Absolutely brilliant… I think mainly the—just checking the draining, checking the—because your drains are in for quite a while afterwards. Your bandaging, things like that. Wounds. Just tending to wounds.”* (Female, breast, short-term survivor, #14)

Other participants valued support provided by a McGrath Breast Cancer Nurse and six participants reported receiving nurse-led, structured, healthy-lifestyle, supportive or clinical trial follow-up via telephone. One of these programs was cancer-specific (Healthy Living After Cancer), while others were more general, for example run by private health funds. Whatever the scheme, participants who had been offered and accepted it, reported that nurse-led lifestyle-focused telephone support was highly valued.
  *“I was in a program that—the Healthy Living after Cancer… That really got me up and going and started walking. I liked it because it was good when you had the weekly phone calls… you were more accountable for it week to week… she was really good…she always rang when she said she was ringing.”* (Female, breast, short-term survivor, #17)

**PART B: How post-treatment quality of life-related needs could be better addressed**

Six overarching themes and a number of subthemes emerged from the data on how rural cancer survivors’ post-treatment quality of life-related needs could be better addressed.

### 3.5. Openness to Telephone and Face-to-Face Modalities of Quality of Life Support

Participants were consistently keen to engage with telephone or face-to-face services for programs designed to help improve their quality of life.

- An element of ***human interaction/connection*** is valued, particularly face-to-face.
  *“I still think it’s got to be the face-to-face stuff too… you need that connection.” * (Female, breast, long-term survivor, #13)

b. However, participants also expressed familiarity with and acceptance of accessing support via ***telephone***.
  *“I think perhaps over the phone. I’m not a person that has a difficulty with talking about anything in front of another person, so I wouldn’t have any problems talking over a phone, but I certainly would rather talk to somebody than have something on a keyboard or something, yeah.”* (Female, colorectal, long-term survivor and carer, #8)

### 3.6. Need Services to Reach Out to Us, Rather Than Waiting for Us to Reach Them

Due to a reluctance to seek help, participants consistently expressed valuing services that reached out to offer support to them proactively, rather than them having to initiate contact with the service themselves.

*“So maybe if these things do happen automatically at the end… Maybe not even give you the option because you think, treatment’s finished, I’m going home, it’s all going to be okay, so I probably—I may not have opted in for it anyway and then you get home and go, well actually, it’s not quite what I expected…Whereas, if someone’s calling you, you have to answer the phone don’t you?”* (Female, cervical, long-term survivor, #16)

*“Yeah, I think… the young rural male is very unaccepting or–or un-unwilling to ah go out and seek that help because they think, oh nah, I’m tough, I’m strong enough, I can do that. But, nah, just don’t [laughs], you’ve got to tell blokes that—insist you’ve gotta—you’ve gotta get help.”* (Male, testicular, short-term survivor, #4)

- A subtheme that emerged from some participants was the notion that they also recognize the ***value in being pressed to talk about emotional issues***, because it is not something they are used to doing.
  *“…and someone who had the ability to pry information out of you, too, because you do put up a barrier—well I do, put up barriers.”* (Female, colorectal/ lymphoma short-term survivor and carer of long-term prostate cancer survivor, #21)

### 3.7. Barriers to Accessing Support via Internet-Based Programs Still Exist

Challenges associated with poor internet connections, access to a computer and lack of familiarity with the internet or computers were consistently raised by participants as barriers to accessing internet-based programs.

*“So I’m way behind with technology. I’m only just learning some things. I’ve only just learnt the other day how to do a multiple text to more than one person… The email thing is—I don’t have my head around that.”*

(Female, breast, short-term survivor, #18)

*“You’d be surprised the aged people, older people, that don’t necessarily have… access to computers or have computers.”*

(Female, lymphoma, short-term survivor, #15)

### 3.8. Continuity of Care Highly Valued

Participants consistently stated that it was preferable for the same person to provide support at each point of contact, so that a relationship was developed, and information did not need to be repeated.

*“So, in the end, I asked them not to ring me anymore… The problem was, more than anything, every time—it was never the same nurse. So there was no continuity and they kept going over the same old questions.”*

(Male, prostate, long-term survivor, #9)

### 3.9. Nurses Are Considered an Appropriate Profession to Deliver Post-Treatment, Quality of Life-Focused Support

Participants consistently stated that they felt nurses were well-placed to support them with the post-treatment issues they experienced, either over the phone or in person, in their local communities.

*“…even the nurses that are here in the–the practice nurses at the surgery, being that local person to help on that… and just to talk rather than… twice a week going to Adelaide or ringing someone in Adelaide. But if it’s through the system here...”*

(Male, prostate long-term survivor and carer of colorectal/ lymphoma short-term survivor, #20)

### 3.10. Telehealth Is a Popular Alternative to Face-to-Face Medical Specialist Appointments as It Reduces the Burden of Travel

Participants value the option of having consultations with their city-based treating specialists via telehealth, due to the reduction in the burden of travel it affords them and their families.

*“We had the, um, video link up with Whyalla which was great ‘cause if mum’s got to go to Whyalla I take a day off work… Well it’s—it’s a two-and-a-half hour drive.”*

(Female, carer of bones/breast long-term survivor, #3)

*“That [telehealth service] was good and to this day I still don’t know why [the medical specialist] stopped it. I did two or three here in [the local] surgery… So just why he stopped it we can’t really find out and I think that’s a crying shame because they are good.”*

(Male, prostate, long-term survivor, #12)

## 4. Discussion

Consistent with previous research on the post-treatment experiences of the broader, non-rural cancer population [30], this study highlighted a range of issues that impacted the quality of life of rural cancer survivors, well beyond the end of treatment. These include both physical health (e.g., fatigue, surgery and medication-related adverse effects) and psychological issues (e.g., fear of cancer recurrence) that often require management. Rural survivors in the present study also expressed frustration with their reduced levels of independence, unmet expectations about their return to pre-cancer quality of life, and their lack of information on how to access support to manage these issues. While these frustrations, particularly with the lack of guidance on post-treatment care, are not unique to rural survivors [31], it is possible that physical distance from metropolitan treatment centres, and rural survivors’ lack of confidence in their local rural health services’ ability to help them address post-treatment issues, may compound these issues in the rural context. Qualitative studies of rural breast [8] and haematological [10,11,12] cancer survivors have highlighted similar concerns about the capacity of rural health services to meet post-treatment needs. These studies also described the strong sense of isolation that survivors experience after leaving their treating team and returning home, a finding that was reflected in the present study.

The findings relating to rural cancer survivors’ lack of knowledge about how to manage post-treatment challenges are consistent with a recent American study that found that 58% of rural cancer survivors lacked information on side effects and symptoms and 54% lacked information on health promotion [32]. Survivorship care plans have been widely recommended to help address these issues [31] and may have particular benefits for rural survivors [32]. However, implementation is sporadic. In the United States, only about 25% of cancer survivors receive a survivorship care plan, and only 62% of rural survivors (versus 78% of urban survivors) report receiving any advice about post-treatment follow up care at all [33], reinforcing the need for improvements in this area of care.

This study builds on the previous, aforementioned qualitative research in this field [8,10,11,12] by including perspectives from rural participants with a broad range of cancer types, both short and long-term survivors, as well as their carers. This study has also extended previous research by examining experiences relating to post-treatment quality of life and medical issues, beyond experiences accessing formal psychosocial care (e.g., [9]). The findings provide new insights into the reasons rural cancer survivors’ may lack confidence in the ability of rural health services to provide the required help. Explanations included the lack of continuity in the tenure of rural GPs, long waiting times to access services, difficulty understanding their GP, concerns about quality of care, concerns about the limited scope of medical services provided by rural hospitals, as well as the lack of availability of quality of life-enhancing services and programs in these settings. Similar concerns have been reported by rural people after intensive, city-based treatment for other serious health challenges such as, organ transplants (e.g., [34]), suggesting that these rural challenges are unlikely to be cancer-specific. Addressing rural health workforce shortages has been a priority for governments since the early 1990s in Australia [35] and needs to continue to be, in addition to the adoption of innovative models of care, if the significant inequality in rural/urban health outcomes are to be addressed.

Given these issues of rural access, it is unsurprising that we found that most support for post-treatment quality of life- concerns was provided by family and friends. Consistent with findings from other tumour-specific rural studies (e.g., [10]), this practical lay support is highly valued, particularly because survivors often experience fatigue and other treatment-related side effects that limit their ability to perform daily tasks. In addition, many rural survivors who participated in the present study reported receiving support from nurses, who either visited their homes (e.g., to dress wounds) or spoke to them via phone and delivered structured programs and support. These forms of support were greatly appreciated.

Equally consistent with previous work [8,10,11], participants in this study reported challenges associated with returning to metropolitan centres for ongoing cancer surveillance and follow-up care. This comes with logistical challenges and significant financial costs associated with time away from work and/or family—particularly when medical appointment scheduling is completed without taking rural survivors’ travel requirements into consideration and/or a travel companion is required. These findings are consistent with other, tumour specific study findings (e.g., [8,9,10,11,12]), and suggest that all rural cancer survivors may experience unique challenges in the post-treatment period. Careful consideration of these issues by all urban health professionals (e.g., when scheduling appointments) would be beneficial.

Many of the novel insights identified in this research relate to how rural cancer survivors and their carers believe their post-treatment needs related to quality of life—could be better addressed. We found that rural Australian cancer survivors are generally open to accessing telephone and face-to-face services and programs designed to assist them with improving their quality of life, and their carers are willing to support them to do so. This finding is supported by a recent, international systematic review that concluded that rural *breast* cancer survivors are interested in participating in, and are generally satisfied with, programs designed to provide them with psychosocial support, weight management and survivorship knowledge, and that they can be successfully delivered remotely via telephone or videoconference [36]. The present study highlights that for programs to be impactful in rural Australia, services may need to reach out to rural survivors (rather than waiting for survivors to make contact). Participants also highlighted that dealing with post-treatment emotional issues was something they knew they could benefit from assistance with, but they would need to be pressed to speak about. These findings may reflect the attitudinal barriers to help-seeking that are known to operate in rural contexts, particularly when it comes to accessing support for emotional or mental health issues [37], even for cancer [6]. Pascal et al. [9] explained that rural cancer survivors’ self-imposed barriers to accessing psychosocial care may be due to their high visibility in small communities, and describe this as ‘the void’. They also speculated that ‘the void’ may be exacerbated by rural health professionals deliberately maintaining emotional distance from their patients and clients, due to the multi-faceted nature of the relationships they have with them [9]. The aforementioned workforce shortages and rural health professionals’ associated lack of time, may exacerbate this issue. However, where possible, making the discussion of the emotional impact of cancer (e.g., adjustment, fear of cancer reoccurrence) a standard part of any new survivorship program or service, may play a valuable role in helping to normalise these sorts of challenges and facilitate important discussions in rural contexts.

Despite the increasing use of online platforms to deliver interventions aiming to improve the quality of life of cancer survivors around the globe [25], and the great potential for them to provide care directly to rural survivors in the privacy and comfort of their homes, another important insight from this study is that barriers to accessing support via internet-based programs still exist for some rural cancer survivors in Australia. Although 77% of Australians living in remote or very remote areas have internet access, this access is not uniformly distributed and those over 65 years are least likely to have it. Moreover, this is the age group most likely to be diagnosed with cancer. This finding of digital inequity and insecurity is consistent with results from a recent Australian systematic review in which it was concluded that digital interventions are best used only as an adjunct service to other forms of support for rural melanoma survivors post-treatment [38]. It also aligns with recent recommendations for careful consideration of digital health literacy levels to be given when implementing digital technology in cancer care [39].

Most rural survivors who participated in the present study expressed familiarity with and acceptance of accessing support via telephone. The value of human interaction and connection was also highlighted, as was the importance of continuity of care so that information does not have to be repeated and rapport can be built. Nurses were consistently considered an appropriate profession to deliver post-treatment, quality of life-focused support (including the highly desired emotional support), possibly because of rural survivors’ familiarity with this profession, and nurses’ scope of practice across both health and mental health-related issues. Services delivered by nurses may be associated with less stigma than services delivered by mental health professionals such as psychologists. This is an encouraging finding because delivering support via nurses, particularly in more remote settings, is likely to be a feasible approach. It also aligns with previous research that concludes that nurses are well-placed to provide education, care planning and support to rural cancer survivors [10,40].

It should be noted that the nurse-led telephone-based initiative Healthy Living After Cancer (HLAC) program [41]—which has been found to significantly improve physical activity, dietary intake, weight, quality of life, cancer-related side-effects and fear of cancer recurrence in Australian cancer survivors—has great scope, because it meets many of the preferred methods for delivery of support reported by participants in this study. However, a limitation of the HLAC program is that it has limited focus on mental health. This may be an area of expansion that would be particularly valued by rural survivors. There are also other rural models of support that are disease specific (e.g., [19]) that could be trialled with broader tumour groups, given the similarities in experiences between tumour groups found in this study. In turn, this would increase the feasibility of such models for use in less-densely populated regions.

Finally, unsurprisingly, telehealth was found to be a popular alternative to face-to-face medical specialist appointments because of the reduced burden of travel. The value of telehealth for rural people affected by cancer is now well established [42] but uptake has been slow. The data reported here were collected before the COVID-19 pandemic, which has led to greater adoption of telehealth practices by clinicians [43] and may have helped resolve this issue. However, there is likely to still be value in consistently reminding urban-based clinicians of the particular benefits of telehealth for their rural patients, given its potential to help address disparities in cancer survival between rural and urban patients [7] as well as improve quality of life. It should also be noted that while rural cancer survivors in this study clearly valued telehealth consultations with their treating doctors, only a few considered the internet to be an acceptable method for delivery other types of support (e.g., psychosocial programs). This probably reflects participants’ perceptions of web-based support being delivered in the form of static websites, rather than interactive web-resources or teleconferenced consultations. Further research into perceptions of teleconferenced quality of life-enhancing programs in the Australian rural cancer survivor population would be useful, especially given that there is emerging evidence they are acceptable in the rural American context (e.g., [44]).

## 5. Conclusions

Many of these findings on the post-treatment experiences of rural cancer survivors are consistent with those reported by cancer survivors universally, regardless of their place of residence. Other findings on post-treatment challenges aligned with issues that have been highlighted in previous research with rural people with breast and haematological cancers, suggesting these are largely rural-specific, rather than tumour-specific issues. Without increased attention to the specific challenges and intervention preferences of rural cancer survivors outlined in this paper, the benefits of efforts directed towards helping cancer survivors improve their post-treatment quality of life are unlikely to be experienced by those living beyond the boundaries of urban centres, and inequality in long-term survival is likely to continue to grow.

## Figures and Tables

**Table 1:** Interview topics.

Basic cancer-related questions: personal cancer or carer history, cancer type, date of diagnosis, cancer status, treatment types, treatment location, treatment completion
The experience of completing cancer treatment and returning to the rural community, and challenges during this time (for the patient) What patient did to cope/maximise quality of life Patient’s engagement with behaviour (e.g., exercise, healthy eating, ongoing surveillance) to prevent cancer from coming back or to detect recurrences early
Knowledge, use and perceptions of existing services Difficulties in accessing existing services Factors considered when choosing support services Unmet social, emotional, practical, spiritual and psychological needs of the patient How could existing services better meet these needs? How could new services meet these needs? What would these new services look like, and why would they be useful? How needs changed over time

**Table 2:** Demographic and cancer history characteristics of the sample.

Participant Characteristics		n
Age in years	M = 60.55, Range = 35–78	
Gender		
Female		14
Male		8
Marital status		
Single/never married		1
Married/living with a partner		18
Widowed		1
Separated/divorced		2
Education level		
Finished primary school		3
Finished high school		7
Trade certificate, apprenticeship, diploma/certificate from a college or TAFE		8
Degree/diploma from a university		3
Postgraduate degree		1
Level of remoteness (Accessibility/Remoteness Index of Australia)		
Inner regional		4
Outer regional		11
Remote		4
Very remote		3
Participant-reported site of disease		
Breast		8
Colorectal/bowel		4
Lung		1
Lymphoma		3
Prostate		7
Other		7
Participant-reported time between treatment completion and interview		
<12 months		6
1–2 years		1
2–5 years		7
>5 years		8
Participant-reported cancer treatments received		
Chemotherapy		12
Hormone therapy		7
Radiotherapy		15
Surgery		11
Other		1

Note. Total ≥22 as some participants reported multiple sites and treatments. Carers reported medical characteristics on behalf of the person for whom they care.

## Data Availability

The data are not publicly available.
